# Parkinson Disease: An Evolutionary Perspective

**DOI:** 10.3389/fneur.2017.00157

**Published:** 2017-05-01

**Authors:** Pedro J. Garcia-Ruiz, Alberto J. Espay

**Affiliations:** ^1^Department of Neurology, Fundacion Jimenez Diaz, Madrid, Spain; ^2^James J. and Joan A. Gardner Family Center for Parkinson’s Disease and Movement Disorders, University of Cincinnati, Cincinnati, OH, USA

**Keywords:** Parkinson disease, evolutionary approach, life expectancy, dopaminergic field, physical activity

## Abstract

There are two central premises to this evolutionary view of Parkinson disease (PD). First, PD is a specific human disease. Second, the prevalence of PD has increased over the course of human history. Several lines of evidence may explain why PD appears to be restricted to the human species. The major manifestations of PD are the consequence of degeneration in the dopamine-synthesizing neurons of the mesostriatal neuronal pathway. It is of note the enormous expansion of the human dopamine mesencephalic neurons onto the striatum compared with other mammals. Hence, an evolutionary bottle neck was reached with the expansion of the massive nigrostriatal axonal arborization. This peculiar nigral overload may partly explain the selective fragility of the human dopaminergic mesencephalic neurotransmission and the unique presence of PD in humans. On the other hand, several facts may explain the increasing prevalence of PD over the centuries. The apparently low prevalence of PD before the twentieth century may be related to the shorter life expectancy and survival compared to present times. In addition, changes in lifestyle over the course of human history might also account for the increasing burden of PD. Our hunter-gatherers ancestors invested large energy expenditure on a daily basis, a prototypical physical way of life for which our genome remains adapted. Technological advances have led to a dramatic reduction of physical exercise. Since the brain release of neurotrophic factors (including brain-derived neurotrophic factor) is partially exercise related, the marked reduction in exercise may contribute to the increasing prevalence of PD.

Parkinson disease (PD), the second most prevalent neuropsychiatric neurodegenerative disorder (1–5), increases exponentially with aging (3, 4). While PD likely represents a syndrome of several molecular subtypes, with a small proportion arising from well-defined genetic abnormalities (1, 5), the data accumulated suggest two common denominators: alpha synuclein accumulation into cytoplasmic Lewy bodies and dopamine deficiency as the casualty of neuronal loss in the nigrostriatal neurons (1, 5). The convergence of diverse biological processes, including genetic, environment, and behavioral factors in the development of PD suggests that evolutionary processes may explain in part the vulnerability to this form of neurodegeneration (6). From a phylogenetic standpoint, PD is prevalent among human primates at the top of the evolutionary chain but evidence for its presence in other species is lacking.

There are two central premises to the evolutionary view of PD. First, PD is a specific human disease. Despite the importance of animal models to the understanding of potential pathogenic mechanism in PD (5), there is no naturally occurring parkinsonism in non-human species: PD is a specific human disease. Second, the prevalence of PD has increased over the course of human history (7), and probably even over the last century (8). Several converging evolutionary theories can explain these observations.

## PD is Specifically Human

Many neurological diseases can be found in non-human mammals (9–12) both acquired and hereditary (such as myelopathy, brain tumors, epilepsy, muscular dystrophy, and narcolepsy, to mention a few). However, Alzheimer disease and PD are considered specific to *Homo sapiens* (13–15). While there are useful animal models of PD including MPTP and alpha-synuclein-overexpressing transgenic mouse models, which may recapitulate important clinical features of the human disorders (5, 14), especially in aged monkeys (16, 17), no spontaneous akinetic-rigid syndrome is known to occur in wild mammals including non-human primates.

Several lines of evidence serve to explain why PD appears to be restricted to the human species. The major motor manifestations of PD are the consequence of degeneration in the dopamine-synthesizing neurons of the mesostriatal neuronal pathway (1, 13). In particular, the dopaminergic fields in the striatum of neurons from the substantia nigra pars compacta (SNpc) can be far more extensive than those of other neurotransmitter types (18). The elegant anatomical studies of Matsuda and colleagues illustrated that the magnitude of the SNpc-to-neostriatum relationship whereby the axon of a single tyrosine hydroxylase-positive dopaminergic neuron in the rat occupies up to 6% of the volume of the striatum (18). Conversely, the arborization of the human mesostriatal neurons occupies a much larger volume of striatum compared to other vertebrates. Vernier and colleagues suggested that the peculiar phenotype of the dopamine mesencephalic neurons, selected during vertebrate evolution, and reshaped in the human lineage, has rendered these neurons particularly prone to oxidative stress (13). Bolam and Pissadaki also stressed the enormous expansion of the human dopamine mesencephalic neurons onto the striatum compared with other mammals (19, 20). Some figures are impressive: the volume of the striatum has increased by approximately 300-fold from rats (20 mm^3^) to humans (6,280 mm^3^), but the *number* of dopaminergic neurons in the SNpc has increased by only 32-fold (rats, 12,000; humans, 382,000) (19). Thus, human dopamine nigral neurons must give rise to axons 10 times the size and 10 times the number of synapses compared to rats (19, 20). Pissadaki and Bolam elegantly proposed that this axonal architecture creates high-energy demands on dopamine-producing nigral neurons to maintain cell functions including the propagation of action potentials (19, 20). As these authors suggested, the nigral neurons are on the edge of an energetic catastrophe (19). Hence, an evolutionary bottle neck was reached with the expansion of the massive (and unmyelinated) nigrostriatal axonal arborization. This peculiar nigral overload may partly explain the selective fragility of the human dopaminergic mesencephalic neurotransmission and the unique presence of PD in humans (13, 19, 20).

Finally, it is relevant to note that this phylogenetically overloaded system needed to regulate very complex motor behaviors. Motor control among mammalians became progressively more sophisticated as hominids developed such skilled motor behaviors as stone tools manufacturing (21). The basal ganglia are known to be critical for the acquisition, improvement, and sustainability of skilled motor behaviors (22, 23).

## PD Prevalence has Increased Over the Course of Human Story

The “official” history of PD is quite recent. PD was named after the contribution of James Parkinson in 1817 (24). Although James Parkinson was first in bringing attention to this particular disease, aspects of the disease had been described by Galen, Sylvius, Juncker, and Cullen (24). Early artistic descriptions of PD can also be found in painting (25, 26). In any case, until the second part of the nineteenth century, PD was virtually unknown to physicians. It is relevant to ask why a motorically *obvious* disease such as PD was unnoticed until relatively recently. A plausible explanation is that PD was actually rare. James Parkinson described only six patients, and over the next century, even experienced neurologists such as Gowers and Wilson, and others did not report a large number of parkinsonian patients (27–30), Gowers only studied 80 cases in detail: “*Of eighty cases, of which I have notes, fifty were men and thirty women*” (27) and Wilson included in his textbook (1940) a table with 383 patients “… *combining the collection of Erb, Peterson, Bychowski, Ruherman and Manschot*” (28). Over the last few decades, the incidence and prevalence seem to have increased according to several authors (4, 8, 31), although for others, PD may have reached a nadir (31–34).

The apparently low prevalence of PD before the twentieth century may be related to the shorter life expectancy and survival compared to present times (4, 8, 31, 34). In addition, historical changes in lifestyle might also account for the increasing burden of these diseases. As O’Keefea and colleagues noted, our ancestors, surviving as hunter-gatherers, required large energy expenditures on a daily basis, and this way of life represented the prototypical physical activity regimen for which our genome adapted (35). O’Keefea et al. and Mattson suggested that technological advances (from the agricultural revolution to the industrial revolution and to the recent digital revolution) have led to progressive but dramatic reduction in physical exercise and overall activity in daily routines (35, 36). Critically, however, our inherent exercise capabilities and needs, selected after thousands of years of evolution, remain essentially unchanged as compared with those of our ancestors (35). That physical exercise is important for everyone is supported by the fact that many chronic ailments and age-related diseases including PD are associated with sedentarism (34–37). Of course, it is impossible to know exactly the collective level of daily physical exercise achieved by our stone age ancestors, but O’Keefea and colleagues suggested that the energy expenditure on physical activity of hunter-gatherers was at least four times that of the modern humans (35).

Physical activity has not only proven beneficial in preventing but also attenuating the motor and cognitive developments of PD (34, 37–40). Moderate or vigorous physical activity is associated with a >30% reduction in PD risk (40). In addition, a protective role of physical activity on PD risk is also supported by animal models (41, 42). Physical exercise seems to be one of the few practical strategies with neuroprotective potential across all neurodegenerative diseases (35, 37, 43). The putative neuroprotective benefits of physical exercise may be explained by several mechanisms including the production of neuroprotective factors such brain-derived neurotrophic factor and glial cell-derived neurotrophic factor (42–45). Vigorous exercise should, therefore, be accorded a central place in the primary prevention and secondary management of PD (35, 37–40, 43).

**Figure 1:**
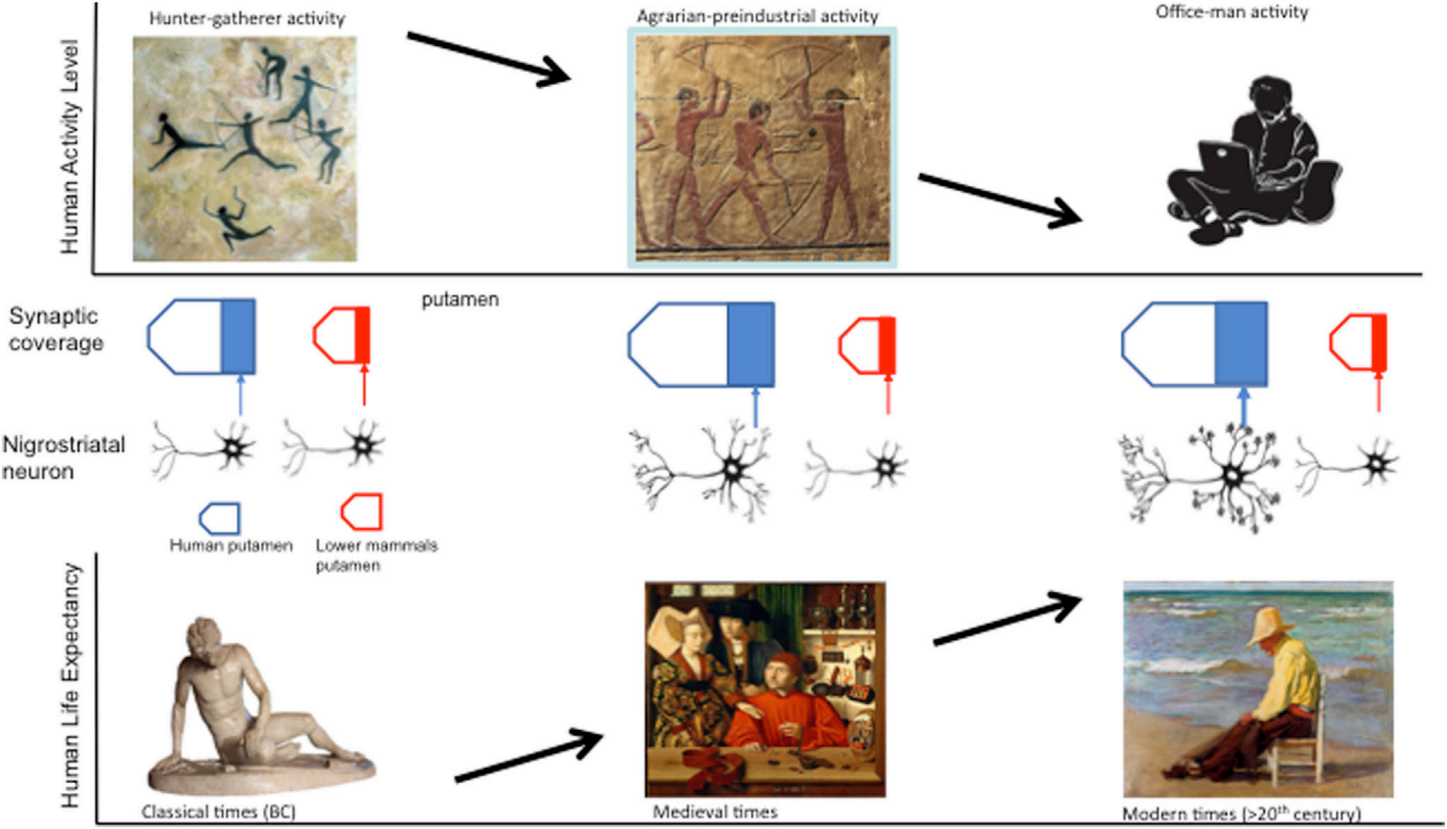
**Evolutionary and behavioral variables that may contribute to the presence of Parkinson disease: increasing nigroestriatal arborization and synaptic putaminal coverage from lower species to humans, decreasing activity levels, and increasing human life span over the course of millennia**.

In summary, important evolutionary clues may explain the specificity of PD to human species, as shown in Figure 1:

1. The phenotype of the dopamine mesencephalic neurons, selected during vertebrate evolution and reshaped in the human lineage, has rendered these neurons particularly prone to oxidative stress, and thus, to the selective neurodegeneration of PD (13, 18).
2. The size and arborization of theses expanded mesencephalic neurons compared with other vertebrates have made human dopamine nigral neurons vulnerable to an energetic crisis (19, 20).

Finally, other evolutionary concepts may partially explain the increasing presence of PD in our society, including the following:

1. The apparently low prevalence of PD before the twentieth century may be related to a shorter life expectancy and survival (4, 8, 31, 34).
2. Changes in lifestyle over the course of human history might also account for the increasing burden of PD. Our hunter-gatherers ancestors invested large energy expenditure on a daily basis, a prototypical physical way of life for which our genome remains adapted (35, 36).
3. Technological advances have led to a dramatic reduction of physical exercise for daily routines (35, 36).
4. Since the brain release of neurotrophic factors is partially exercise-related (44, 45), the reduction in exercise at a societal level may contribute to the increasing prevalence of PD and other neurodegenerative disorders in our era of digital revolution.

## Author Contributions

PG-R: conception and design, interpretation of data, drafting the submitted material, and critical review. AE: conception and design, interpretation of data, critical revision, and supervision.

## Conflict of Interest Statement

PG-R received research support from Allergan and UCB, personal compensation as a consultant/scientific advisory board from Italfarmaco, Britannia, Bial, and Zambon and speaking honoraria from Italfarmaco, UCB, Zambon, Allergan, and Abbvie. AE has received grant support from NIH, Great Lakes Neurotechnologies, and The Michael J. Fox Foundation; personal compensation as a consultant/scientific advisory board member for Abbvie, TEVA, Impax, Merz, Acadia, Cynapsus, Lundbeck, and USWorldMeds; publishing royalties from Lippincott Williams & Wilkins, Cambridge University Press, and Springer; and honoraria from Abbvie, UCB, USWorldMeds, Lundbeck, Acadia, the American Academy of Neurology, and the Movement Disorders Society. He serves as Associate Editor of the Journal of Clinical Movement Disorders and on the editorial board of Parkinsonism and Related Disorders.
